# Effects of receiving renal biopsy on the prognosis of chronic kidney disease patients with impaired renal function

**DOI:** 10.1186/s12882-023-03097-2

**Published:** 2023-03-15

**Authors:** Tianyi Zhang, Xiaoqian Yang, Minfang Zhang, Wenyan Zhou, Yan Jin, Hang Zhou, Yin Zhou, Qin Wang, Shan Mou

**Affiliations:** Department of Nephrology, Molecular Cell Lab for Kidney Disease, Shanghai Peritoneal Dialysis Research Center, Ren Ji Hospital, Uremia Diagnosis and Treatment Center, Shanghai Jiao Tong University School of Medicine, Shanghai Jiao Tong University, Shanghai, 200127 China

**Keywords:** Chronic kidney disease, Renal biopsy, Prognosis

## Abstract

**Background:**

Impaired renal function was not a recognized indication for renal biopsy. The effects of receiving renal biopsy on the renal functional prognosis for chronic kidney disease (CKD) patients with impaired renal function need to be explored.

**Methods:**

This study retrospectively enrolled 300 renal function impaired CKD patients in Renji Hospital from January 2015 to December 2017, 150 of them received percutaneous renal biopsy while the others did not. The endpoint was ≥ 50% estimated glomerular filtration rate (eGFR) decline from baseline or development of end-stage renal disease (ESRD). Kaplan-Meier analysis with log-rank test was performed to compare the renal survival probability between patients receiving renal biopsy or not. Univariate and multivariate analysis with Cox regression were conducted with predictors of poor renal outcomes in the study cohort.

**Results:**

The median follow-up period was 37.6 months. During the follow-up period, the eGFR of the biopsy group increased from 52.2 ± 14.4 to 67.4 ± 37.8 ml/min/1.73 m², but decreased from 55.3 ± 17.1 to 29.8 ± 19.1 ml/min/1.73 m² in the non-biopsy group. Patients who received renal biopsy had significantly higher renal survival probability (P < 0.001). Cox regression analysis revealed that 24-hour urine protein excretion (24 h UPE) more than 1 g/d was an independent predictor for poor renal outcomes in the non-biopsy group but not in the renal biopsy group (HR = 1.719, P = 0.040).

**Conclusion:**

CKD patients with impaired renal function are recommended to receive renal biopsy to make pathological diagnoses, especially for those with the 24-hour urine protein excretion more than 1 g/d.

**Supplementary Information:**

The online version contains supplementary material available at 10.1186/s12882-023-03097-2.

## Introduction

Chronic kidney disease (CKD) has been gradually becoming one of the most important public health burdens worldwide. A study enrolling 47,204 participants showed that the overall prevalence of CKD was 10.8% (10.2-11.3%) [1]. While the ERA-EDTA registry annual report 2012 revealed that among patients undergoing renal replacement therapy (RRT), 15% of them had no specific renal pathological diagnosis, which meant no precise treatments targeting their primary kidney disease [2].

Renal biopsy is the gold standard to diagnose renal diseases. However, there is currently poor consensus about proper indications of this procedure [3]. Most of the nephrologists agreed that renal biopsy should be performed in patients with nephrotic syndrome (NS), proteinuria more than 1 g/d, proteinuria, hematuria and acute kidney injury (AKI) for pathological findings to provide information for treatment alteration and prediction of prognosis [4–7]. Impaired renal function was not a recognized indication for renal biopsy. Because subjects with impaired renal function may have a higher risk of bleeding, also the renal tissue obtained by biopsy may not give enough information for diagnosis and change of treatment if chronic renal damages prevail [8]. However, previous studies found that skipping renal biopsy in patients with renal impairment may miss treatable interstitial nephritis [9], which may progress to ESRD quickly without proper treatment. Systematic diseases including systemic lupus erythematosus (SLE), vasculitis and allergic purpura, may affect the kidney function and cause renal impairment. And renal biopsy is a crucial method to evaluate the activity and severity of the diseases. Thus, it is important to get a better knowledge of the baseline and pathological characteristics of the cohort with renal impairment.

Nowadays most nephrologists empirically decided whether the renal biopsy is needed for CKD patients with impaired renal function, at the same time, relied on the levels of other characteristics including proteinuria, hematuria et al. As no studies have focused on the pathological diagnosis of the population with impaired renal function, and their renal prognoses after biopsy were still unclear. Meanwhile, as the gold standard to diagnose renal diseases, no studies have evaluated the effects of receiving renal biopsy on the long-term renal function for CKD patients. In the current study, we intended to explore the effects of receiving renal biopsy on the prognosis of CKD patients with impaired renal function, and to find the independent predictors for poor renal outcomes in renal function impaired CKD patients who did not receive renal biopsy, thus providing clinical evidence for better management of CKD patients with impaired renal function.

## Methods

### Study population

Chronic kidney disease (CKD) patients with impaired renal function from January 2015 to December 2017 in Renji Hospital were retrospectively reviewed. The inclusion criteria were as follows: (1) aged at 18 or older; (2) diagnosed as CKD; (3) with baseline eGFR less than 90ml/(min·1.73 m²); (4) without acute kidney injury (AKI). The exclusion criteria were as follows: (1) patients with incomplete data; (2) follow-up period less than 6-month; (3) patients with contraindications of percutaneous renal biopsy [10], including small kidneys or end stage renal disease (ESRD), inability to provide informed consent, multiple bilateral cysts, uncorrectable bleeding diathesis, severe hypertension which cannot be controlled with antihypertensive medications, hydronephrosis, urinary tract infection, pyelonephritis, or perirenal abscess/infection, horseshoe kidney and uncooperative patient or inability to follow instructions during biopsy.

### Definitions

CKD is defined as the abnormalities of kidney structure or function, presenting for more than 3 months, with implications for health [11]. Patients with impaired renal function were defined according to the Kidney Disease Outcomes Quality Initiative (K-DOQI) guidelines: GFR ≥ 90ml/min/1.73m^2^ (normal renal function), GFR < 90ml/min/1.73m^2^ (renal impairment) [12]. Estimated glomerular filtration rate (eGFR) was calculated by the Modification of Diet in Renal Disease abbreviated equation for Chinese patients: eGFR = 186×(Scr/88.4)^−1.154^ × age^− 0.203^ (×0.742, female) [13].

To rule out the influence of AKI-induced decrease in eGFR on the diagnosis of CKD, some patients with predominantly acute injury on renal biopsy pathology were excluded prior to formal statistical analysis. For patients receiving no renal biopsy, the serum creatinine and eGFR levels of patients were recorded retrospectively for at least 3 months prior to inclusion to exclude patients with AKI.

### Endpoints

The endpoint was ≥ 50% eGFR decline from baseline or development of end-stage renal disease (defined as eGFR < 15ml/min/1.73m^2^, need for dialysis or renal transplantation).

### Data recollection and patients follow-up

Clinical parameters taken into consideration for each patient were as follows: preoperative medical condition (hypertension and/or diabetes mellitus), baseline serum creatinine, eGFR-MDRD, 24-hour urine protein excretion, haemoglobin, serum albumin and hematuria (defined as ≥ 4 red blood cells per high power field of view). The pathological diagnoses of patients in the renal biopsy group were also collected. Treatment regimens used by the patients after renal biopsy or enrollment were recorded through the hospital information system (HIS). Baseline parameters were tested within three days to the operation in the renal biopsy group and when patients meet the inclusion criteria for the first time in the non-renal biopsy group. Follow-up time was considered as the interval between eGFR ≤ 90ml/(min·1.73 m²) or renal biopsy and the last outpatient visit, or the incident to the endpoints.

### Statistical analysis

Baseline characteristics were retrospectively collected through hospital information system. The statistical analysis was completed with SPSS, version 26.0. Variables were summarized as frequency and percentage for categorical variables, and were employed with the Chi-square test. The normally distributed variables were expressed as means ± standard deviation and compared using a t-test. The non-parametric variables were expressed as the medians with 25th and 75th percentiles and compared using the Mann-Whitney U test. Kaplan-Meier analysis with log-rank test was used for comparing the renal survival probability between patients receiving renal biopsy or not. Univariate and multivariate analysis with Cox regression were conducted with predictors of poor renal outcomes in the study cohort. P<0.05 is considered to be statistically significant.

## Results

### Baseline clinical characteristics

A total of 300 patients with the eGFR less than 90ml/min/1.73m^2^ were finally enrolled into the retrospective study. Among them 150 patients received renal biopsy, 150 patients did not receive renal biopsy (Supplementary Fig. 1). The baseline characteristics for CKD patients with impaired renal function receiving renal biopsy or not are displayed in Table 1. The median follow-up period was 37.6 months. There was no difference in demographic, preoperative medical, and laboratory characteristics between renal biopsy and non-renal biopsy groups. The mean eGFR was 52.2 ± 14.4 ml/min/1.73 m² in the biopsy group compared to 55.3 ± 17.1ml/min/1.73 m² in the non-biopsy group, with a p value showed no significant difference, indicating the baseline renal function was comparable between two groups.

**Table 1:** Characteristics at baseline for patients with impaired renal function receiving renal biopsy or not

Characteristics	Group			P-value
	Total (n = 300)	Renal biopsy (n = 150)	Non-Renal biopsy (n = 150)	
Age (years)	53.0(40.3, 62.0)	52.5(38.0, 60.0)	55.0(42.0, 64.0)	0.090
Male, n (%)	184 (61.3%)	89(59.3%)	95 (63.3%)	0.477
Preoperative medical condition, n (%)				
Diabetes mellitus	50(16.7%)	21(14.0%)	29(19.3%)	0.215
Hypertension	182(60.7%)	88 (58.7%)	94(62.7%)	0.478
Follow-up time (months)	37.6(21.9, 51.8)	41.1(12.78, 54.48)	36.2(24.3, 50.7)	0.951
Serum creatinine (umol/L)	120.0(103.9, 147.0)	124.2(104.0, 160.1)	116.0(103.4, 142.1)	0.102
Haemoglobin (g/L)	132.2 ± 20.4	129.8 ± 15.6	134.7 ± 24.2	0.098
Serum albumin (g/L)	41.0(38.5,43.7)	40.1(38.5, 42.8)	42.4(37.8, 44.7)	0.100
24 h UPE (g/d)	0.94(0.30, 1.99)	1.04(0.36, 2.09)	0.59(0.17, 1.87)	0.083
Hematuria				0.105
No	158	72	86	
Yes	142	78	64	
eGFR (ml/min/1.73m^2^)※	53.8 ± 15.9	52.2 ± 14.4	55.3 ± 17.1	0.100

Data were given as means ± standard deviations or median [25th, 75th ] for continuous features, cases (percentage) for categorical variables

※The estimated glomerular filtration rate as calculated by the Modification of Diet in Renal Disease equation

P value: comparation between renal biopsy and non-renal biopsy group. UPE, urine protein excretion; eGFR, estimated glomerular filtration rate

^2^

### Pathological findings for patients receiving renal biopsy

Among the 150 patients receiving percutaneous renal biopsy, 88 (58.67%) of them were diagnosed as primary glomerular nephropathy (PGN), including IgA nephropathy (IgAN, 31.3%), focal segmental glomerular sclerosis (FSGS, 9.3%), membranous nephropathy (MN, 5.3%), et al. 41 (27.3%) of them had impaired renal function secondary to other systemic diseases, such as lupus nephritis (LN, 10.7%), vasculitis nephropathy (VN, 6.7%), diabetic kidney disease (DKD, 5.3%), Henoch-Schönlein purpura nephritis (HSPGN, 2.0%), hypertensive nephropathy (HN, 1.3%), et al. In addition, 14 (9.3%) patients were diagnosed with tubulointerstitial disease (TID) based on pathological findings (Table 2). Cases and percentages of each specific pathological type were illustrated by column and pie chart in Supplementary Fig. 2. Among all the pathological types, IgAN was the most common pathological diagnosis. Lupus nephritis was the most common secondary glomerular nephropathy (SGN) in our institution.

**Table 2:** Pathological type analysis of patients with impaired renal function who received renal biopsy

Pathological type	Cases, n (%) (150 in total)
**Primary glomerular nephropathy (PGN)**	
IgA nephropathy (IgAN)	47 (31.3%)
Focal segmental glomerular sclerosis (FSGS)	14 (9.3%)
Membranous nephropathy (MN)	8 (5.3%)
Others	19 (12.7%)
**Secondary glomerular nephropathy (SGN)**	
Lupus nephritis (LN)	16 (10.7%)
Vasculitis nephropathy (VN)	10 (6.7%)
Diabetic kidney disease (DKD)	8 (5.3%)
Henoch-Schönlein purpura nephritis (HSPGN)	3 (2.0%)
Hypertensive nephropathy (HN)	2 (1.3%)
Others	2 (1.3%)
**Tubulointerstitial disease (TID)**	14 (9.3%)
**Others**	7 (4.7%)

### Correlation between receiving renal biopsy or not with renal outcomes

**Fig. 1:**
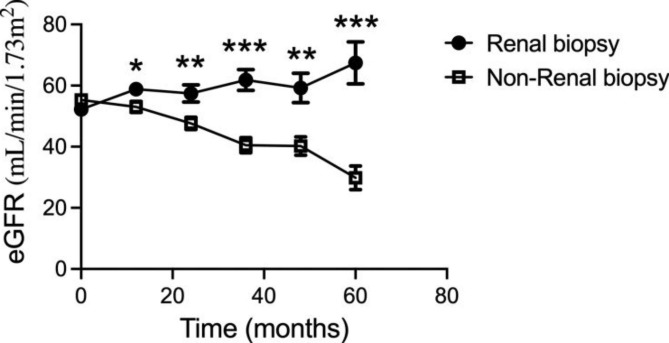
**The eGFR level during follow-up period for CKD patients with impaired renal function receiving renal biopsy or not.** eGFR, estimated glomerular filtration rate

**Fig. 2:**
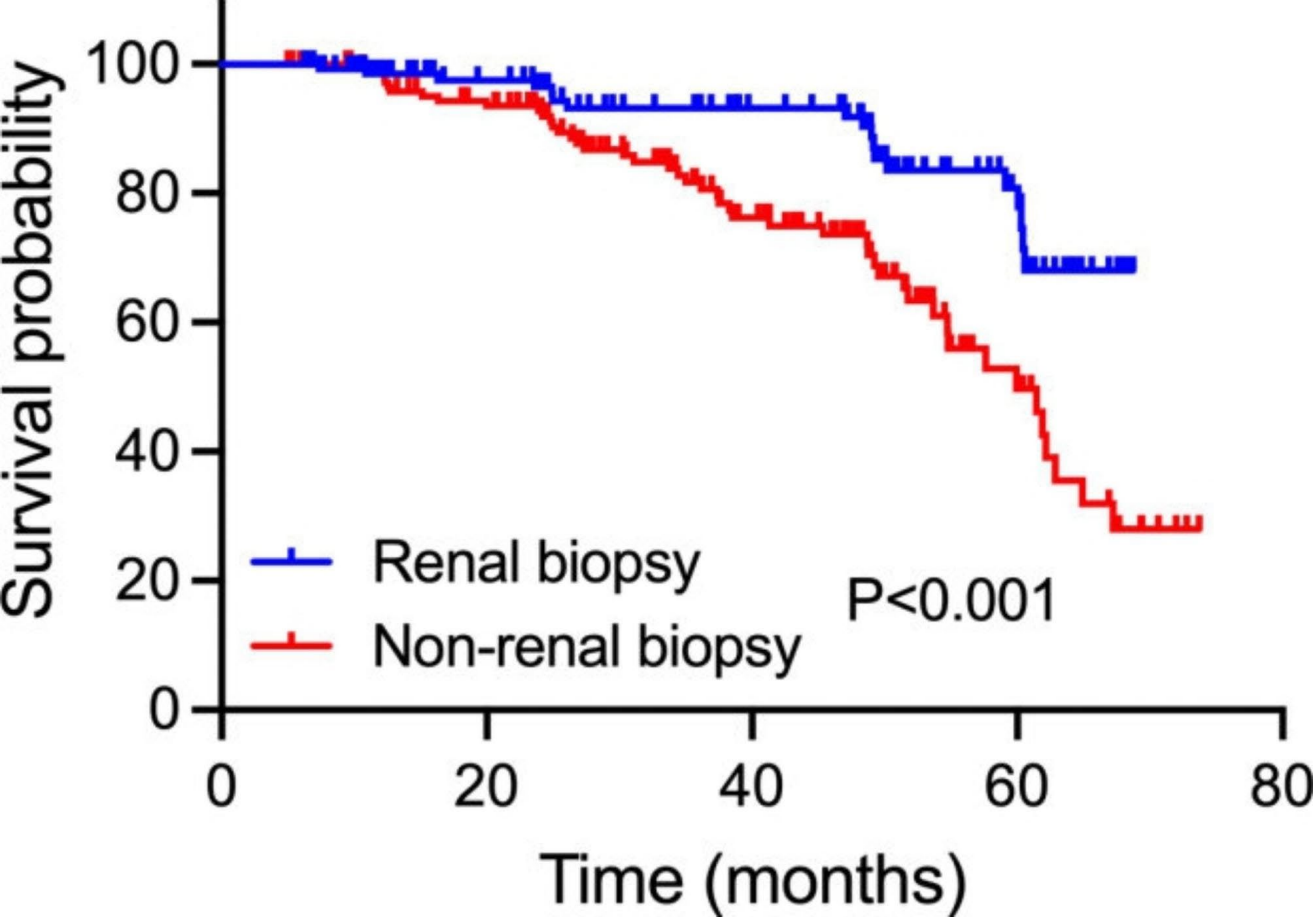
**Association between receiving renal biopsy or not and long-term renal outcomes for CKD patients with impaired renal function.** The blue and red line separately illustrate the survival probability over time for patients receiving renal biopsy or not. Kaplan-Meier analysis with log-rank test revealed a significant difference between groups (P < 0.001)

To determine the effects of renal biopsy on the renal outcomes for patients with impaired kidney function, the eGFR during the 5-year follow-up period was calculated and compared between renal biopsy and non-renal biopsy groups (Table 3). After 1-year follow-up time, the eGFR of patients in the renal biopsy group increased to 58.8 ± 24.8 ml/min/1.73 m², indicating improvement of short-term overall renal function after renal biopsy. While the eGFR decreased from 55.3 ± 17.1 to 53.0 ± 19.9 ml/min/1.73 m² in non-renal biopsy group (p = 0.039). For patients receiving renal biopsy, the eGFR increased to 67.4 ± 37.8 ml/min/1.73 m² five years after the renal biopsy, which was a 29.1% increase from the baseline. Conversely, the eGFR was 29.8 ± 19.1 ml/min/1.73 m² (p < 0.001) in non-biopsy group after 5-year follow-up, which was a 46.1% decrease from the baseline. Over the 5 years, the mean eGFR showed an overall upward trend in renal biopsy group, while a rapid downtrend in non-renal biopsy group (Fig. 1). For the renal outcomes, 24 (16.0%) patients in renal biopsy group compared to 46 (36.7%, p = 0.003) patients in non-renal biopsy group showed a ≥ 50% decrease of eGFR from baseline or developed ESRD during the follow-up period. Association between receiving renal biopsy or not and long-term renal outcomes for CKD patients with impaired renal function was illustrated in Fig. 2. It is indicated that patients receiving renal biopsy had significantly better renal outcomes in the long-term period compared to patients without pathological diagnoses (P < 0.001).

**Table 3:** The renal outcome for patients with impaired renal function receiving renal biopsy or not

	Renal biopsy (n = 150)	Non-Renal biopsy (n = 150)	P-value
eGFR* during the follow-up period			
1 year	58.8 ± 24.8	53.0 ± 19.9	0.039
2 years	57.4 ± 26.5	47.6 ± 22.1	0.004
3 years	61.8 ± 27.8	40.5 ± 23.8	< 0.001
4 years	59.2 ± 38.1	40.2 ± 24.7	0.001
5 years	67.4 ± 37.8	29.8 ± 19.1	< 0.001
**Endpoints**			
≥ 50% decline from baseline, n (%)	16(10.7%)	44(29.3%)	< 0.001
< 15mL/min/1.73m^2^, n (%)	17(11.3%)	33(22.0%)	0.013
Composite endpoints	24(16.0%)	46 (30.7%)	0.003

Data were given as means ± standard deviations for continuous features and cases(percentage) for categorical features.

* The estimated glomerular filtration rate as calculated by the Modification of Diet in Renal Disease equation.

P value: comparation between renal biopsy and non-renal biopsy group. Composite endpoints: incident of either of the eGFR declined ≥ 50% from baseline or eGFR decreased to < 15mL/min/1.73m^2^ or both during the follow-up time. eGFR, estimated glomerular filtration rate.

### Relationship between treatment regimen and renal outcome in biopsy and non-biopsy group

**Fig. 3:**
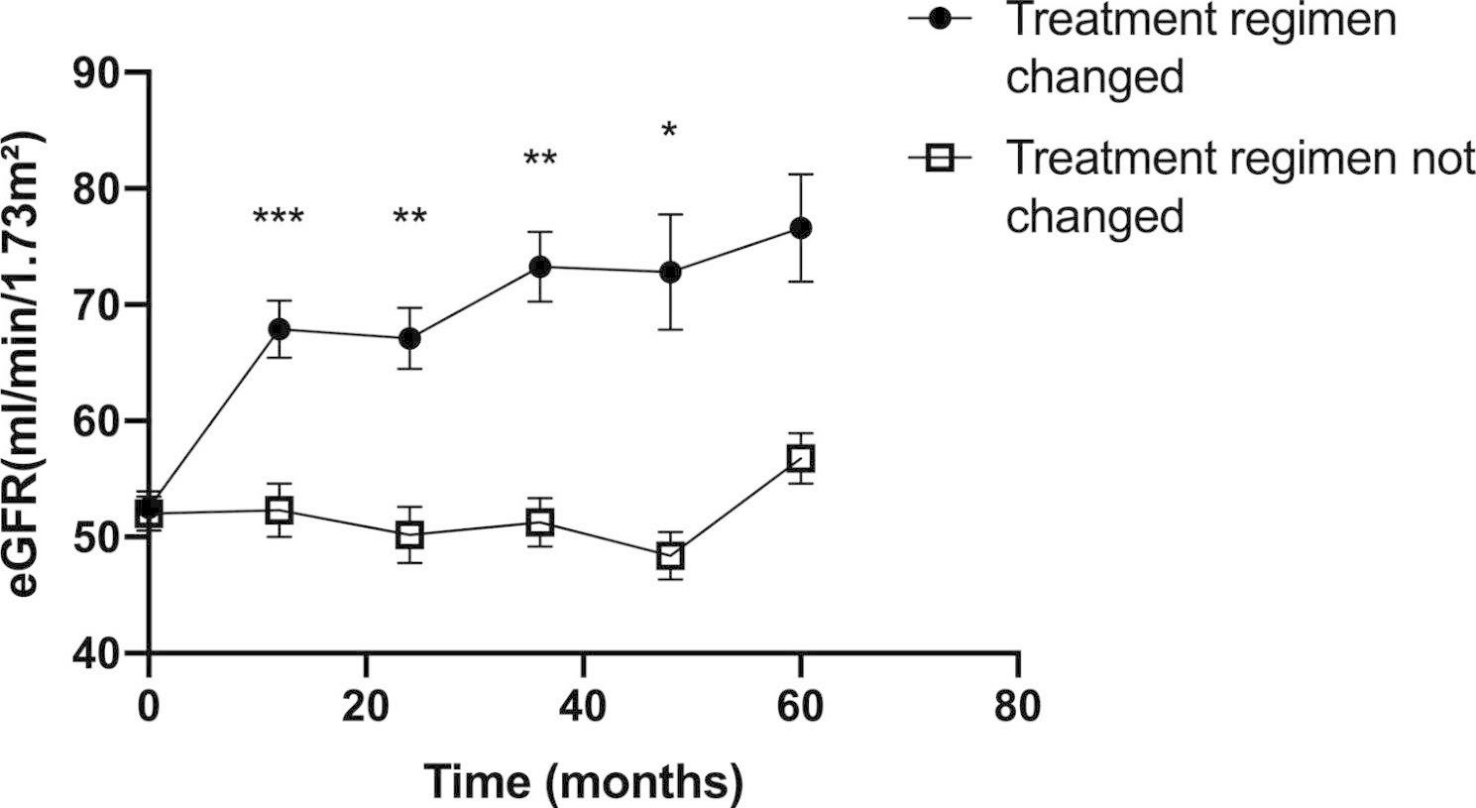
**The eGFR level during follow-up period for CKD patients whose treatment regimen changed or not due to renal biopsy pathology results. eGFR, estimated glomerular filtration rate**

Two independent experienced nephrologists gave treatment regimens based on baseline characteristics of patients before renal biopsy, then compared it with the actual treatment regimen taken by the patients after renal biopsy. If the two regimens were consistent, the patients were assigned to the treatment unchanged group; if the regimens were not consistent, the results of renal biopsy influenced the treatment, and the patients were assigned to the treatment-altered group (Table 4). Figure 3 showed that patients showed an overall increase in eGFR level 5 years after the renal biopsy, regardless of whether the treatment regimen has changed as a result of renal biopsy. However, patients in the treatment-altered group showed a greater increase in eGFR level, and more significant improvement in renal function. The renal outcome for patients treated with and without glucocorticoid, hydroxychloroquine or other immunosuppressive drugs after renal biopsy were also compared (Supplementary Table 1). The results showed a greater increase in eGFR in patients who added glucocorticoid, hydroxychloroquine or other immunosuppressive drugs, but no significant reduction in the rate of endpoint events (Supplementary Fig. 3).

In non-biopsy group, few patients were treated with glucocorticoid or other targeted treatments because there was no clear pathological diagnosis. Renal outcome for patients receiving no renal biopsy treated with angiotensin converting enzyme inhibitor (ACEI) or angiotensin receptor blocker (ARB) were compared (Supplementary Table 2). However, no difference was observed between these two groups (Supplementary Fig. 4).

**Table 4:** The renal outcome for patients whose treatment regimen changed or not due to renal biopsy pathology results

	Treatment-altered group (n = 61)	Treatment unchanged group (n = 89)	P-value
eGFR* during the follow-up period			
1 year	67.90 ± 24.69	52.31 ± 22.88	< 0.001
2 years	67.10 ± 26.45	50.20 ± 24.42	0.002
3 years	73.25 ± 30.27	51.27 ± 20.74	0.001
4 years	72.81 ± 49.65	48.40 ± 20.56	0.010
5 years	76.62 ± 46.49	56.76 ± 21.66	0.155
**Endpoints**			
≥ 50% decline from baseline, n (%)	6 (9.8%)	10 (11.2%)	0.997
< 15mL/min/1.73m^2^, n (%)	6 (9.8%)	11 (12.4%)	0.828
Composite endpoints	9 (14.8%)	15 (16.9%)	0.906

Data were given as means ± standard deviations for continuous features and cases(percentage) for categorical features.

* The estimated glomerular filtration rate as calculated by the Modification of Diet in Renal Disease equation.

P value: comparation between renal biopsy and non-renal biopsy group. Composite endpoints: incident of either of the eGFR declined ≥ 50% from baseline or eGFR decreased to < 15mL/min/1.73m^2^ or both during the follow-up time. eGFR, estimated glomerular filtration rate.

### Predictors for poor renal outcomes

**Fig. 4:**
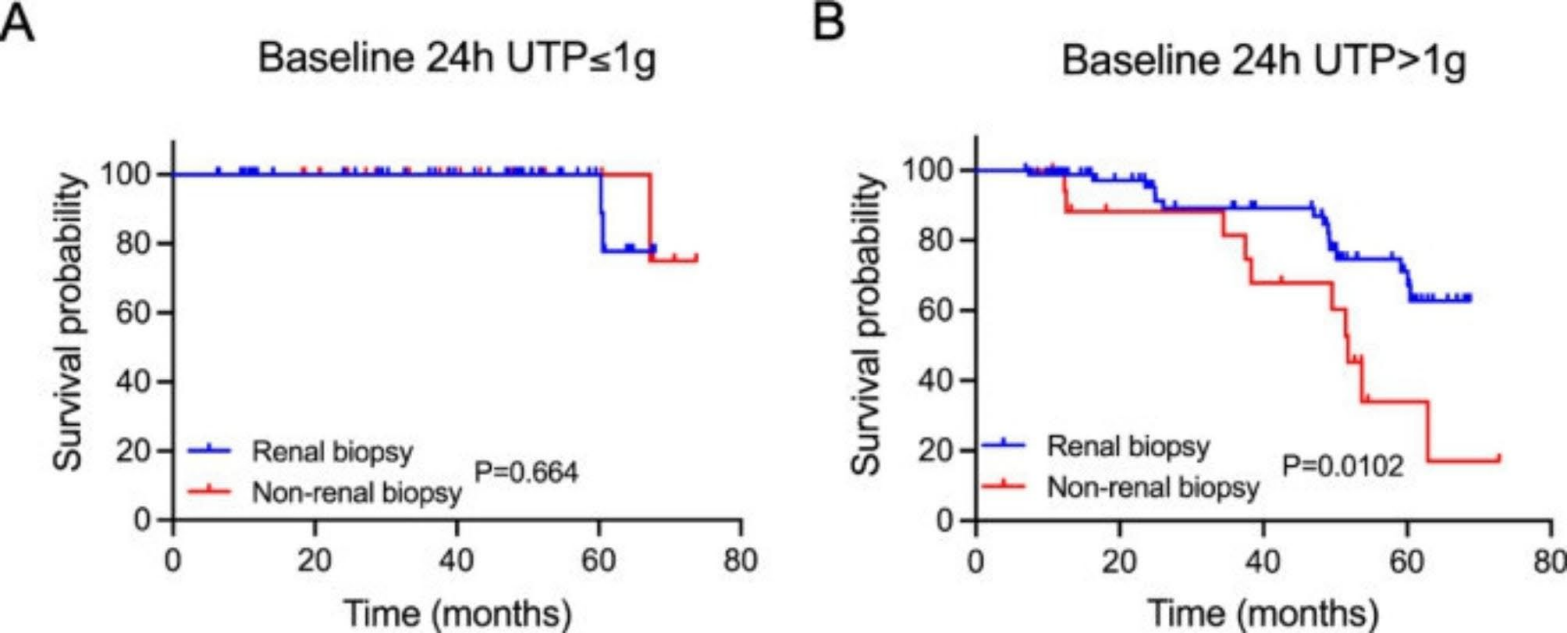
**Association between receiving renal biopsy or not and long-term renal outcomes for CKD patients with impaired renal function stratified by baseline 24 h UTP.** (A) Effects of receiving renal biopsy or not on long-term renal outcomes for patients with baseline 24 h UTP ≤ 1 g/d (P = 0.664). (B) Effects of receiving renal biopsy or not on long-term renal outcomes for patients with baseline 24 h UTP > 1 g/d (P = 0.0102). The blue and red line separately illustrate the renal survival probability over time for patients receiving renal biopsy or not

CKD patients with impaired renal function who received renal biopsy or not had different renal outcomes during the follow-up period, so we did univariate and multivariate cox regression analysis separately for these two groups (Table 5). For the renal biopsy group, complicating diabetes mellitus was an independent predictor for poor renal prognosis (HR = 18.741, p < 0.001). While for the non-renal biopsy group, the 24-hour urine protein excretion (24 h UPE) may predict poor renal outcomes independently (HR = 1.719, p = 0.040). Hypertension, diabetes mellitus, low haemoglobin and hematuria were univariatly associated with poor renal outcomes, but not as independent predictors. As the 24 h UPE was an independent predictor for the poor renal outcomes in patients without receiving renal biopsy, but not for the renal biopsy group. We further checked the roles of baseline 24 h UPE in the development of renal outcomes with the cut-off value as 1 g/d. Among patients with 24 h UPE less than 1 g/d, there was no significant difference in the survival probability between the renal biopsy group and the non-renal biopsy group during the follow-up period (P = 0.664). While for patients with baseline 24 h UPE more than 1 g/d, patients receiving renal biopsy had a significantly better renal function prognosis compared with patients without pathological diagnoses (P = 0.0102) (Fig. 4). In terms of pathologic types for the cohort with 24 h UPE more than 1 g/d, IgAN and LN were still the most common pathological types among patients with 24 h UPE > 1 g/d, with 26 (31.3%) cases and 13 (15.7%) cases respectively in 83 patients (Table 6). Moreover, compared with the overall patients receiving renal biopsy, patients with the 24 h UPE more than 1 g/d had slightly higher proportion of patients diagnosed with membranous nephropathy (7.2% vs. 5.3%), and much higher diagnostic rate of lupus nephritis (15.7% vs. 10.7%) (Supplementary Fig. 5).

**Table 5:** The univariate and multivariate Cox regression analysis for predictors of poor renal outcome in patients with impaired renal function receiving renal biopsy or not

Characteristics	Univariate analysis				Multivariate analysis		
	HR	95% CI	P		HR	95% CI	P
**Renal biopsy group**							
Age	1.019	0.980–1.059	0.350				
Gender (Male)	0.333	0.117–0.948	0.039				
Hypertension	0.589	0.166–2.095	0.414				
Diabetes mellitus	18.986	3.676–98.059	< 0.001		18.741	3.627–96.830	< 0.001
GC/HCQ/ISD	0.767	0.334–1.764	0.533				
Haemoglobin	0.990	0.960–1.022	0.533				
Serum albumin	0.819	0.615–1.091	0.172				
Hematuria	1.348	0.298–6.095	0.698				
24 h UPE	1.088	0.991–1.195	0.076				
eGFR in baseline (mL/min/1.73m2)	0.979	0.947–1.102	0.219				
**Non-Renal biopsy group**							
Age	0.988	0.965–1.012	0.323				
Gender (Male)	1.086	0.594–1.984	0.789				
Hypertension	3.089	1.379–6.916	0.006				
Diabetes mellitus	4.464	2.433–8.190	< 0.001				
Haemoglobin	0.983	0.967-1.000	0.046				
ACEI/ARB	0.788	0.378–1.644	0.525				
Serum albumin	0.976	0.879–1.085	0.656				
Hematuria	3.847	1.588–9.320	0.003				
24 h UPE	1.848	1.261–2.708	0.002		1.719	1.025–2.883	0.040
eGFR* in baseline mm2)	0.971	0.951–0.991	0.005				

HR, hazard ratio; 95% CI, 95% confidence interval; GC, glucocorticoid; HCQ, hydroxychloroquine; ISD, immunosuppressive drugs; ACEI, angiotensin converting enzyme inhibitor; ARB, angiotensin receptor blocker; UPE, urine protein excretion; eGFR estimated glomerular fraction rate.

* The estimated glomerular filtration rate as calculated by the Modification of Diet in Renal Disease equation.

**Table 6:** Pathological type analysis of renal biopsy results for renal function impaired patients with baseline 24 h UTP more than 1 g/d

Patients with renal biopsy	Overall (150 in total) Cases, n (%)	24 h UPE > 1 g (83 in total) Cases, n (%)
**Primary glomerular nephropathy (PGN)**		
IgA nephropathy (IgAN)	47 (31.3%)	26 (31.3%)
Focal segmental glomerular sclerosis (FSGS)	14 (9.3%)	8 (9.6%)
Membranous nephropathy (MN)	8 (5.3%)	6 (7.2%)
Others¶	19 (12.7%)	10 (12.0%)
**Secondary glomerular nephropathy (SGN)**		
Lupus nephritis (LN)	16 (10.7%)	13 (15.7%)
Vasculitis nephropathy (VN)	10 (6.7%)	4 (4.8%)
Diabetic kidney disease (DKD)	8 (5.3%)	5 (6.0%)
Henoch-Schönlein purpura nephritis (HSPGN)	3 (2.0%)	3 (3.6%)
Hypertensive nephropathy (HN)	2 (1.3%)	1 (1.2%)
Others§	2 (1.3%)	1 (1.2%)
**Tubulointerstitial disease (TID)**	14 (9.3%)	3 (3.6%)
**Others**※	7 (4.7%)	3 (3.6%)

UPE, urine protein excretion.

¶ Other primary glomerular nephropathy besides IgAN, MN and FSGS;

§ Other secondary glomerular nephropathy besides LN, VN, DKD, HSPGN and HN;

※ Other pathological diagnose besides PGN, SGN and TID.

## Discussion

We demonstrated that CKD patients with impaired renal function who received renal biopsy had better long-term renal outcomes compared to patients without receiving renal biopsy. And baseline 24 h urine protein excretion more than 1 g/d was an independent predictor for poor renal outcomes in patients without specific pathological diagnosis, but not for patients receiving renal biopsy, indicating that renal biopsy should be strongly recommended for CKD patients with impaired renal function, especially for those with relatively mass urine protein excretion.

Kidney biopsy is the gold standard for the diagnosis of pathological kidney diseases since the early 1950s. However, it is an invasive procedure with potential risks, and is recommended only when the pathological findings may affect treatments or provide information for prognosis [14]. Impaired renal function (elevated serum creatine level or decreased eGFR) has been seen as a symbol of chronic renal damage, and renal tissue obtained from biopsy may not give enough information for accurate diagnosis if chronic damage (tubulointerstitial fibrosis, glomerulosclerosis, arteriosclerosis) prevails [4]. As a result, in previous studies indicating the protective effects of renal biopsy, the change of treatments or improvement of renal outcomes commonly resulted from the biopsy diagnosis in patients with acute kidney injury and nephrotic syndrome, less commonly in those with chronic impaired renal function [9]. However, the major managements for CKD patients with impaired renal function were mainly limited to supportive treatments including blood pressure control, use of ACEI/ARB, restriction of the salt and protein intake, glycemic control and prevention of complications [11]. The above supportive treatments were not specifically targeted therapies, which cannot effectively defer the progression of the disease in some cases.

In the current study, we retrospectively reviewed a cohort of 300 CKD patients with the impaired renal function (eGFR less than 90ml/min/1.73 m²), at the same time, without the contraindications of percutaneous renal biopsy. Among them 150 patients received renal biopsy, while the others did not. We found the mean eGFR showed an overall upward trend after receiving renal biopsy for CKD patients with impaired renal function, while deteriorated rapidly for those without receiving renal biopsy. Moreover, patients who received renal biopsy had significantly better renal functional prognosis than those without pathological diagnoses during the follow-up period. The treatment regimens were altered in 61 of 150 (40.67%) patients received renal biopsy, this group of patients had better renal outcomes than those whose treatment regimens were not altered by the results of renal biopsy. These results confirmed that receiving renal biopsy is beneficial for the renal functional survival for CKD patients with impaired renal function by providing more information for the determination of treatment options. The beneficial effects of receiving renal biopsy should be attributed to the identification of pathological diagnoses which could benefit from the directed therapy, which often involves hormones and immunosuppressants [10]. Moreover, receiving renal biopsy is also crucial for evaluating the activity and severity of some renal diseases such as LN, which is closely correlated with the treatment options [15]. In contrast, patients in the non-biopsy group treated with or without ACEI/ARB showed no significant differences in renal outcomes, partially because patients requiring ACEI/ARB have high baseline UPE levels and are inherently at greater risk of kidney disease progression.

In the current study, baseline 24 h UPE turned out to be an independent predictor for poor renal outcomes in patients who did not receive renal biopsy, while not being a risk factor for patients receiving renal biopsy. A study enrolled 638,150 adults suggested that proteinuria of increasing severity is associated with a faster rate of renal function decline, regardless of baseline eGFR [16]. After analyzing data from 28 cohorts including 693,816 participants, Coresh et al. [17] found that in individuals with baseline albumin creatine ratio (ACR) of 300 mg/g or higher, a 30% decrease in ACR over 2 years was estimated to confer a more than 1% absolute reduction in 10-year risk of ESRD, indicating that the decrease of 24 h UPE had protective effects on the long-term renal outcomes for CKD patients. Our results suggested that renal biopsy was highly recommended for patients with a baseline 24 h UPE of 1 g/d or higher, as well as for patients with hypertension, DM, anemia and hematuria. While for patients with a 24 h UPE less than 1 g/d, there was no significant difference for renal survival probability between biopsy and non-biopsy group. However, a retrospective study revealed that SLE patients with low-level proteinuria may have significant lupus- or non-lupus-related kidney diseases with management implications, suggesting that current guidelines are supposed to expand renal biopsy indications to include isolated proteinuria of any grade [18]. Although the current study indicated that 24 h UPE less than 1 g/d was not associated with poor renal outcomes for patients without receiving renal biopsy, the study was limited by its relatively small sample size as well as short follow-up period, with only few patients coming to endpoints in the cohort of patients with 24 h UPE less than 1 g/d. Studies with large amounts of patients are needed to provide more accurate instructions.

For our study, IgAN and lupus nephritis were the most common primary and secondary glomerular nephropathy in the cohort, not only for the overall cohort patients receiving renal biopsy but also for patients with 24 h UPE more than 1 g/d. The pathological diagnoses for IgAN patients are helpful for the classification and selection of therapy. For example, studies suggested that IgAN patients with endocapillary hypercellularity had an improved outcome if treated with corticosteroids [19]. Moreover, IgAN patients with crescents in < 25% of glomeruli identify those at risk of a poor renal outcome if not treated with immunosuppression [20]. It is indicated that if not receiving renal biopsy, IgAN patients with pathological features as above cannot get the targeted therapy, thus leading to the rapid progression of IgAN. As for LN, the biopsy findings have been used to classify and subgroup LN, which is significant for obtaining the accurate diagnosis to make treatment decisions and predicting prognosis for patients with LN [21]. A common perception is that the severity of histology is positive associated with the level of proteinuria. However, a study analyzed the renal biopsy results of 38 SLE patients with 24 h UPE less than 500 mg/d, and found 95% of them were diagnosed as class III, IV, or V LN, whereas only 5% were class II LN, indicating the great discordance between clinical and histologic findings for LN [22]. Although some non-invasive biomarkers may have the ability to rack histologic activity and chronicity, differentiate proliferative from non-proliferative LN, and identify the presence and severity of specific pathologic lesions, none of them have been validated in independent LN populations [23]. Above all, it can be concluded that the treatment decisions and prognosis of LN were much more reliable to renal biopsy results than other pathologic types.

This study has certain value in guiding the treatment process for CKD patients with impaired renal function. According to our results, CKD patients with the eGFR less than 90 ml/min/1.73 m² and proteinuria more than 1 g/d are highly recommended to receive renal biopsy. Patients with renal insufficiency but with low-level proteinuria (24 h UPE ≤ 1 g/d) can choose not to receive renal biopsy at the time being, but the regular follow-up is highly needed. Meanwhile, the renal biopsy need to be re-considered when patients develop 24 h UPE > 1 g/d, AKI or abnormalities in serum markers that may suggest systemic diseases.

There are several limitations to our study. Firstly, the study cohort was under-represented in the general population as only patients in one center were included. Besides, patients with missing data were deleted from the cohort, to some extent having caused the loss of information. Secondly, the sample size and follow-up period were relatively not enough for this study, as the patients with baseline 24 h UPE less than 1 g/d coming to the endpoints were quite few to make further subgroup analysis. Finally, the selection bias existed in this study, especially for the patients without receiving renal biopsy. As most of these patients only had outpatient clinic visit instead of the hospitalization experience, making most of them with limited information. We excluded patients with renal biopsy contraindications from the study cohort. However, some patients without receiving renal biopsy owing to renal biopsy contraindications might be enrolled into the final 300 patients restricted by the limited information. Prospective studies or even RCT studies are needed to verify our conclusions.

## Conclusion

CKD patients with impaired renal function are recommended to receive renal biopsy for pathological diagnoses to instruct the treatments, especially for those with a relatively high level of proteinuria.

## Electronic supplementary material

Below is the link to the electronic supplementary material.

Supplementary Material 1

## Data Availability

Any inquiries towards the datasets generated and/or analyzed during the current study can be directed to the corresponding authors.

## Declarations

### Experimental protocol approval

All experimental protocol were approved by Shanghai Jiaotong University School of Medicine Affiliated Renji Hospital.
