# Supplementary material for: Effects of receiving renal biopsy on the prognosis of chronic kidney disease patients with impaired renal function
Source: BMC Nephrol. 2023 Mar 15;24:56. doi: 10.1186/s12882-023-03097-2 (PMC10018988; doi:10.1186/s12882-023-03097-2)
Supplement: Supplementary file 1 — Supplementary Material 1 [file 12882_2023_3097_MOESM1_ESM.docx]

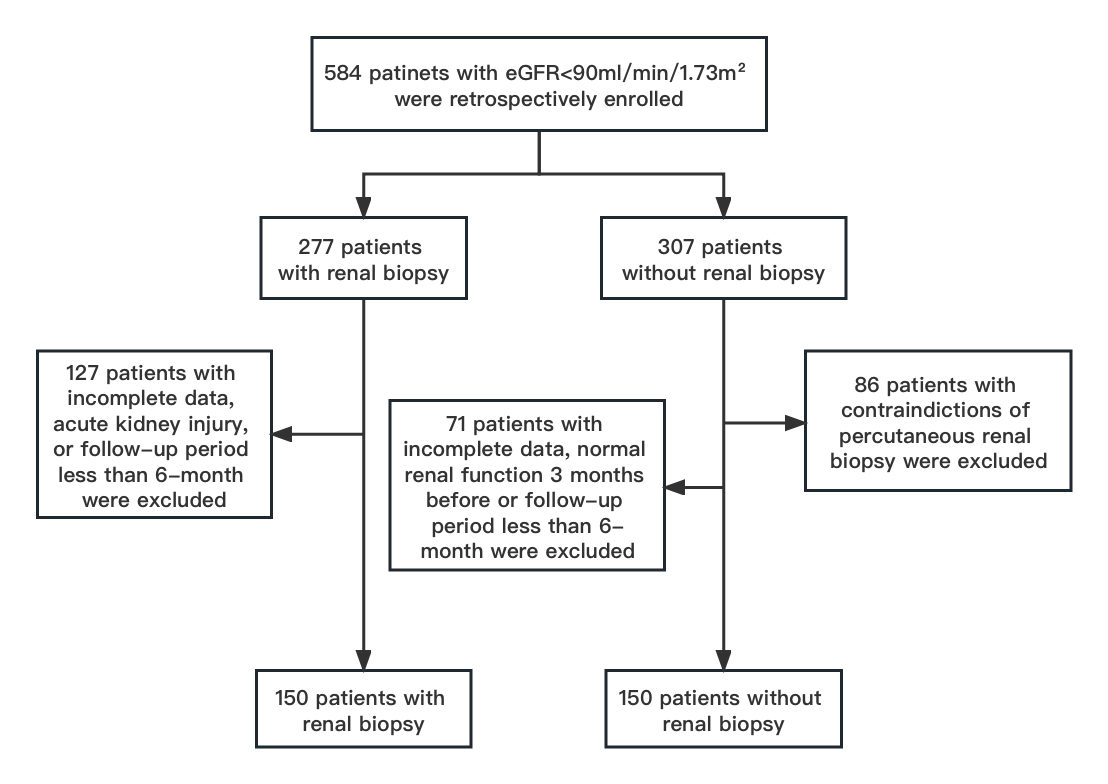


**Supplementary Figure 1. Flow diagram of the study design.** A total of 300 CKD patients with eGFR less than 90ml/min/1.73m^2^ were enrolled into the study. Among them 150 patients received renal biopsy, 150 patients did not receive renal biopsy. eGFR, estimated glomerular filtration rate.


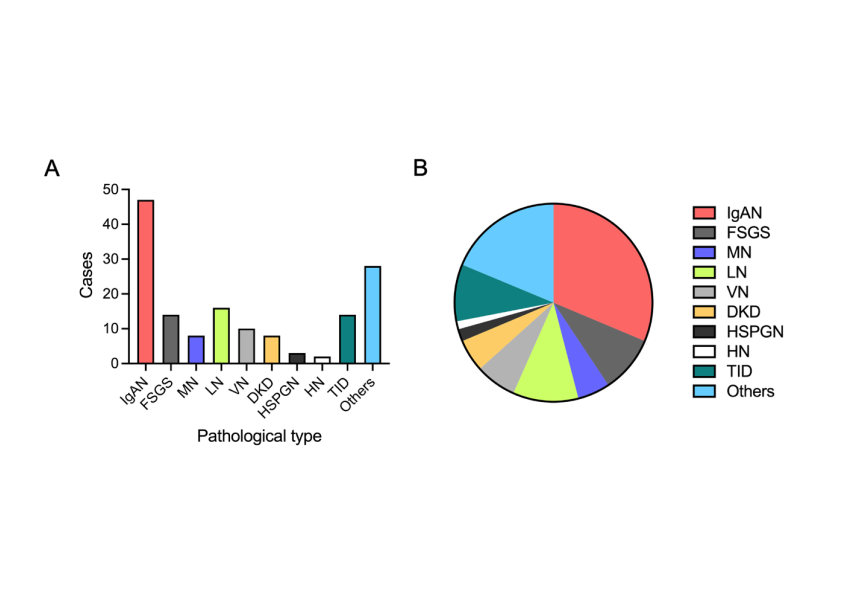


**Supplementary Figure 2. Pathological type analysis of CKD patients with impaired renal function who received renal biopsy.** (A) Cases of each specific pathological type. (B) Percentages of each specific pathological type. IgAN, IgA nephropathy; FSGS, focal segmental glomerular sclerosis; MN, membranous nephropathy; LN, lupus nephritis; VN, vasculitis nephropathy; DKD, diabetic kidney disease; HSPGN, Henoch-Schönlein purpura nephritis; HN, hypertensive nephropathy; TID, tubulointerstitial disease.

**Supplementary Table 1. The renal outcome for patients treated with and without glucocorticoid, hydroxychloroquine or other immunosuppressive drugs after renal biopsy**

|  | **GC/HCQ/ISD group**  **(n=85)** | **Non-GC/HCQ/ISD group**  **(n=64)** | **P-value** |
| --- | --- | --- | --- |
| **eGFR* during the follow-up period** | | | |
| 1 year | 63.99±23.86 | 51.67±24.49 | 0.005 |
| 2 years | 62.46±26.49 | 50.00±25.13 | 0.029 |
| 3 years | 69.17±29.42 | 50.62±21.21 | 0.007 |
| 4 years | 67.37±44.71 | 47.68±22.20 | 0.043 |
| 5 years | 73.81±43.00 | 56.20±24.53 | 0.225 |
| **Endpoints** |  |  |  |
| ≥50% decline from baseline, n (%) | 8 (9.4%) | 8 (12.5%) | 0.737 |
| <15mL/min/1.73m^2^, n (%) | 9 (10.6%) | 7 (10.9%) | 1.000 |
| Composite endpoints | 13 (15.3%) | 10 (15.6%) | 1.000 |

Data were given as means±standard deviations for continuous features and cases(percentage) for categorical features.

* The estimated glomerular filtration rate as calculated by the Modification of Diet in Renal Disease equation.

P value: comparation between renal biopsy and non-renal biopsy group. Composite endpoints: incident of either of the eGFR declined ≥50% from baseline or eGFR decreased to <15mL/min/1.73m^2^ or both during the follow-up time. eGFR, estimated glomerular filtration rate; GC, glucocorticoid; HCQ, hydroxychloroquine; ISD, immunosuppressive drugs.


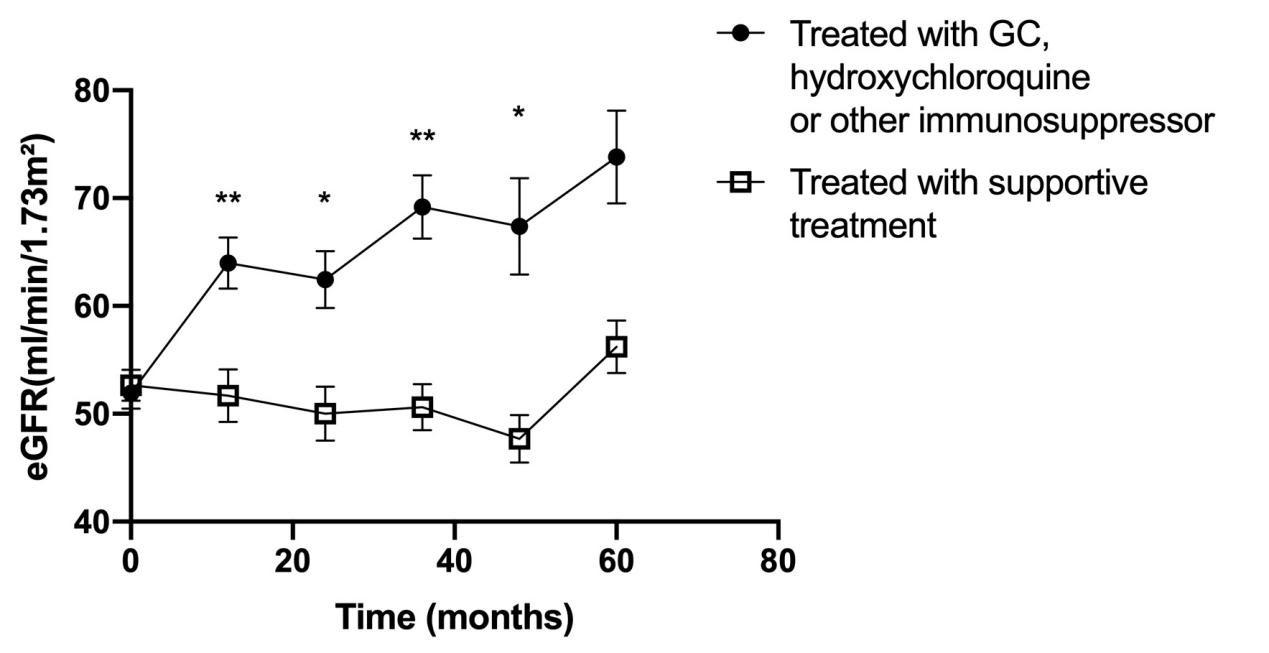


**Supplementary Figure 3. The eGFR level during follow-up period for CKD patients treated with and without glucocorticoid, hydroxychloroquine or other immunosuppressive drugs after renal biopsy.** eGFR, estimated glomerular filtration rate; GC, glucocorticoid.

**Supplementary Table 2. The renal outcome for patients receiving no renal biopsy treated with and without ACEI/ARB**

|  | **ACEI/ARB group**  **(n=85)** | **Non-ACEI/ARB group**  **(n=64)** | **P-value** |
| --- | --- | --- | --- |
| **eGFR* during the follow-up period** | | | |
| 1 year | 54.92±19.46 | 52.55±20.14 | 0.573 |
| 2 years | 52.96±20.12 | 46.16±22.43 | 0.161 |
| 3 years | 41.79±23.93 | 40.11±23.93 | 0.786 |
| 4 years | 38.03±25.64 | 41.11±24.47 | 0.659 |
| 5 years | 32.26±21.29 | 29.16±18.93 | 0.729 |
| **Endpoints** |  |  |  |
| ≥50% decline from baseline, n (%) | 9 (24.3%) | 34 (30.1%) | 0.643 |
| <15mL/min/1.73m^2^, n (%) | 6 (16.2%) | 27 (23.9%) | 0.453 |
| Composite endpoints | 9 (24.3%) | 36 (31.9%) | 0.508 |

Data were given as means±standard deviations for continuous features and cases(percentage) for categorical features.

* The estimated glomerular filtration rate as calculated by the Modification of Diet in Renal Disease equation.

P value: comparation between renal biopsy and non-renal biopsy group. Composite endpoints: incident of either of the eGFR declined ≥50% from baseline or eGFR decreased to <15mL/min/1.73m^2^ or both during the follow-up time. eGFR, estimated glomerular filtration rate; ACEI, angiotensin converting enzyme inhibitor; ARB, angiotensin receptor blocker.


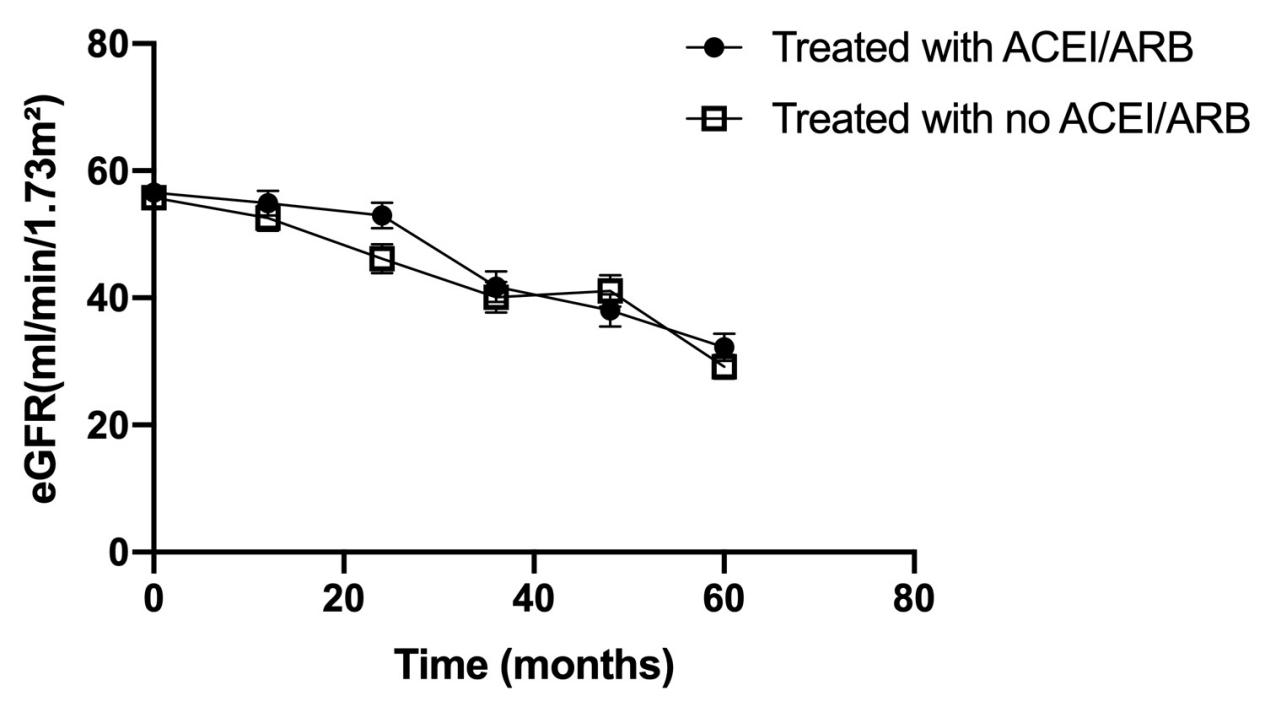


**Supplementary Figure 4. The eGFR level during follow-up period for CKD patients receiving no renal biopsy treated with and without ACEI/ARB.** eGFR, estimated glomerular filtration rate; ACEI, angiotensin converting enzyme inhibitor; ARB, angiotensin receptor blocker.


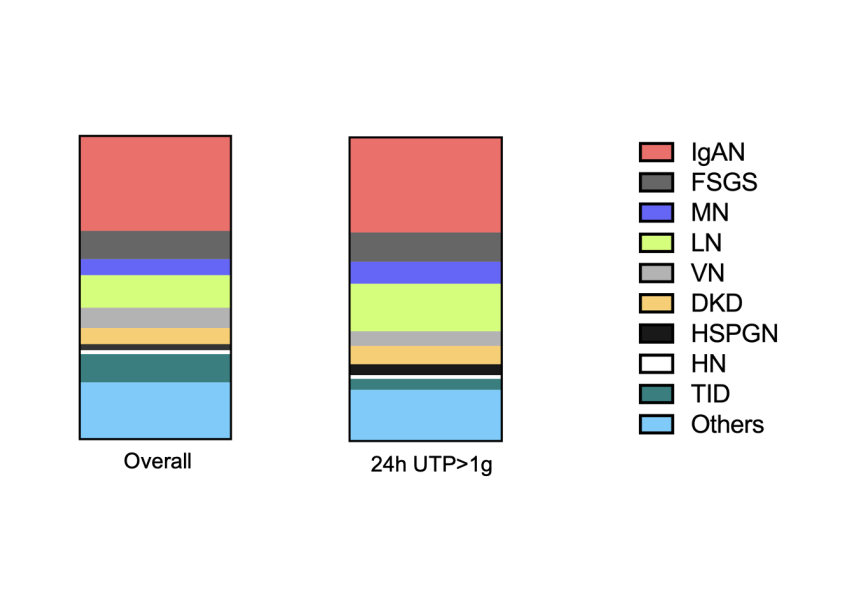


**Supplementary Figure 5. The pathological type ratio comparison between renal function impaired patients with baseline 24h UTP more than 1g/d and overall patients with impaired renal function.** Left column: the percentages of each specific pathological type for overall patients with impaired renal function. Right column: the percentages of each specific pathological type for renal function impaired patients with baseline 24h UTP more than 1g/d. IgAN, IgA nephropathy; FSGS, focal segmental glomerular sclerosis; MN, membranous nephropathy; LN, lupus nephritis; VN, vasculitis nephropathy; DKD, diabetic kidney disease; HSPGN, Henoch-Schönlein purpura nephritis; HN, hypertensive nephropathy; TID, tubulointerstitial disease.
